# Chrysophanic Acid

**Published:** 1877-04

**Authors:** 


Chrysophanic Acid.—This new remedy, of great efficacy in ring-worm, will soon be familiar to our pharmacists. Mr. A. W. Postans, of London, who first prepared it, states that the easiest, simplest, and by far the best method of making it into an ointment is to dissolve the acid in hot fat. Two drachms will dissolve in one ounce of lard, but this is very concentrated. The hot ointment should then be transferred to a mortar, and rubbed down till cold.

If to each ounce of ointment so prepared two drops of otto of roses be added, a most beautiful preparation results, possessing in an eminent degree the active properties of the acid with the delicate and attractive odor of the rose.—*Ex.*

